# Major bat‐borne zoonotic viral epidemics in Asia and Africa: A systematic review and meta‐analysis

**DOI:** 10.1002/vms3.835

**Published:** 2022-05-10

**Authors:** Shahneaz Ali Khan, Mohammed Ashif Imtiaz, Md Mazharul Islam, Abu Zubayer Tanzin, Ariful Islam, Mohammad Mahmudul Hassan

**Affiliations:** ^1^ Department of Physiology, Biochemistry and Pharmacology Faculty of Veterinary Medicine, Chattogram Veterinary and Animal Sciences University Khulshi Chattogram Bangladesh; ^2^ Department of Animal Resources Ministry of Municipality Doha Qatar; ^3^ EcoHealth Alliance New York New York; ^4^ Centre for Integrative Ecology Deakin University Geelong Campus Victoria Australia; ^5^ Queensland Alliance for One Health Sciences School of Veterinary Science The University of Queensland Queensland Australia

**Keywords:** bat–human interface, bats, epidemic, intermediate host, outbreak, spillover, zoonotic virus

## Abstract

Bats are the natural reservoir host for many pathogenic and non‐pathogenic viruses, potentially spilling over to humans and domestic animals directly or via an intermediate host. The ongoing COVID‐19 pandemic is the continuation of virus spillover events that have taken place over the last few decades, particularly in Asia and Africa. Therefore, these bat‐associated epidemics provide a significant number of hints, including respiratory cellular tropism, more intense susceptibility to these cell types, and overall likely to become a pandemic for the next spillover. In this systematic review, we analysed data to insight, through bat‐originated spillover in Asia and Africa. We used STATA/IC‐13 software for descriptive statistics and meta‐analysis. The random effect of meta‐analysis showed that the pooled estimates of case fatality rates of bat‐originated viral zoonotic diseases were higher in Africa (61.06%, 95%CI: 50.26 to 71.85, *l* ^2^% = 97.3, *p *< 0.001). Moreover, estimates of case fatality rates were higher in Ebola (61.06%; 95%CI: 50.26 to 71.85, *l* ^2^% = 97.3, *p *< 0.001) followed by Nipah (55.19%; 95%CI: 39.29 to 71.09, *l* ^2^% = 94.2, *p *< 0.001), MERS (18.49%; 95%CI: 8.19 to 28.76, *l* ^2^% = 95.4, *p *< 0.001) and SARS (10.86%; 95%CI: 6.02 to 15.71, *l* ^2^% = 85.7, *p *< 0.001) with the overall case fatality rates of 29.86 (95%CI: 29.97 to 48.58, *l* ^2^% = 99.0, *p *< 0.001). Bat‐originated viruses have caused several outbreaks of deadly diseases, including Nipah, Ebola, SARS and MERS in Asia and Africa in a sequential fashion. Nipah virus emerged first in Malaysia, but later, periodic outbreaks were noticed in Bangladesh and India. Similarly, the Ebola virus was detected in the African continent with neurological disorders in humans, like Nipah, seen in the Asian region. Two important coronaviruses, MERS and SARS, were introduced, both with the potential to infect respiratory passages. This paper explores the dimension of spillover events within and/or between bat–human and the epidemiological risk factors, which may lead to another pandemic occurring. Further, these processes enhance the bat‐originated virus, which utilises an intermediate host to jump into human species.

## Graphical abstract

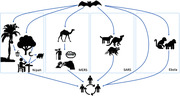

## 1 INTRODUCTION

Bats are the only flying mammals with diverse lifestyles, including long‐distance flying, highly gregarious social structures, long life span and high metabolic activity (Hayman et al., 2013). These animals are the reservoir of many emerging and re‐emerging zoonotic viral pathogens, with a high possibility of this including the current pandemic, SARS‐CoV‐2 (Mackenzie & Smith, 2020). Viral species jumping from bats to other animals happens when a virus obtains the ability to infect and spread among a new host species (Flanagan et al., 2011). In general, the pathogen–host–environment factor interplays a crucial role in viral species jumping from bats to humans. Bat‐associated viruses emerge at the human–wildlife interface, possibly due to changes in the ecology. It is primarily due to human behaviours and activities such as deforestation that cause the changing of landscapes. As a result, bat–human interaction increases and creates viral transmission opportunities to humans (McMichael, 2004). However, a host jump event can happen directly from bats to humans or via a suitable intermediate host, such as livestock, pets or other wildlife (Allocati et al., 2016).

In the last couple of decades, four bat‐born zoonotic viruses took the attention of scientists, Ebola, Nipah, Middle East respiratory syndrome coronavirus (MERS‐CoV) and severe acute respiratory syndrome coronavirus (SARS‐CoV), as they caused several epidemics and high fatalities throughout the world. The species jump of Nipah virus from bats to humans via pigs happened in the last decade (Clayton et al., 2013; Nikolay et al., 2019). MERS‐CoV in 2012 and SARS‐CoV in 2002/2003 were living in bats for an unknown period of time, then utilised palm civets and camels respectively as an intermediate host to overcome the species barrier, got closer to humans and finally resulted in a pandemic explosion (Raoult et al., 2020; Yang et al., 2015). The recent Ebola outbreak in West Africa (Camacho et al., 2014; Olival & Hayman, 2014) exemplifies the need to understand human behaviours and how they may further interact with animal reservoirs and related pathogens. These four viruses all originated from either the Asian or the African continents. The Asian and the African regions have greater wildlife diversity and massive population density that can result in a hub of EIDs (Emerging Infectious Diseases) events frequently (Hassan et al., 2020).

Global efforts to challenge emerging infectious diseases primarily focus on post‐emergence outbreak controls, quarantine and drug and vaccine development (Pike et al., 2014). Understanding human behaviours relating to wildlife contacts and the importance of local community beliefs, regarding wildlife‐originated diseases are often‐neglected (Brian & Mazet, 2018). A greater appreciation of human factors can lead to further understanding of the magnitude of high‐risk behaviours undertaken by community members. Understanding the evolving nature of viruses to the human physiological system is another very important factor for predicting future pandemics.

Case fatality is another important reason to take appropriate measures to control an epidemic (Narayanan, 2020). Overall, the Ebola virus disease (EVD) case fatality is around 50% (World Health Organization, 2021a). It is 40%–75% for Nipah virus disease (NVD) (World Health Organization, 2021b), 11% for SARS (World Health Organization, 2003) and 35% for MERS (World Health Organization, 2018). However, these simple pooling results are inadequate procedures and differ from meta‐analysis as meta‐analysis conduct weight studies and compiles data from subgroups or individual studies (Bravata & Olkin, 2001). Pooled estimates by meta‐analysis are superior to simple pooling estimates. The current study systematically reviewed EVD, MERS, NVD and SARS and analysed pooled case fatality rates. We reviewed bat physiology and immune system, virus spillover, species jump and transmission dynamics of these selected disease pathogens for elicit epidemic or global pandemic.

## 2 METHODOLOGY

### 2.1 Literature search strategy

We used PRISMA (Preferred Reporting Items for Systematic Reviews and Meta‐Analysis) guidelines (Moher et al., 2009) to conduct the systematic review in four steps: database search, evaluating relevant articles, data extraction and summarising. One author conducted the data search and two authors were involved in evaluating the articles and extraction of data individually. It was then compiled by one author and two authors together prepared the data and conducted the meta‐analysis.

**FIGURE 1:**
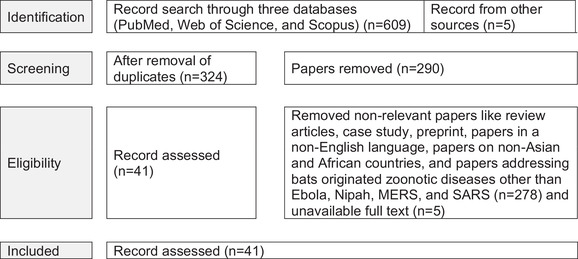
Systematic review PRISMA flow diagram describing the selection of published articles on bat‐originated zoonotic diseases in humans in Asia and Africa, and inclusion/exclusion process used in the study

A literature search on bat‐originated zoonotic diseases in Asia and Africa was performed on 10 July 2021 through PubMed, Scopus and Web of Science (Figure 1). The search covered all the original research articles containing field evidence of bat‐originated zoonotic diseases (SARS, MERS, Ebola and Nipah) among Asian and African countries between 1999 and June 2021. The keywords included for description (Incidence OR Occurrence OR Fatality) AND Continents (Asia OR Africa) AND Outbreaks (SARS‐CoV OR MERS‐CoV OR Ebola OR Nipah). The searches were screened using an advanced search strategy in PubMed [Title/Abstract], Scopus [TITLE‐ABS‐KEY] and Web of Science [Topic].

### 2.2 The search of relevant articles

The search data were processed using EndNote X9 (Clarivate analytics, USA). We identified and excluded the duplicates using EndNote and then studied the title and abstract to find the relevant articles. The relevant articles and the articles that were not clear by the title‐abstract study were subjected to full‐text collection. Only documents published in English were considered for the review (Ahmed, 2017; Ajlan et al., 2014; Alshukairi et al., 2018; Aruna et al., 2019; Arunkumar et al., 2019; Bah et al., 2015; Chadha et al., 2006; Chen et al., 2004; Cheng et al., 2004; Cho et al., 2016; Choe et al., 2017; Chong et al., 2002; Christie et al., 2015; Chua et al., 1999; Cowling et al., 2015; Ding et al., 2003; Dixon et al., 2014; Dowell et al., 1999; Francesconi et al., 2003; Goh et al., 2000; He et al., 2005; Homaira et al., 2010; Hossain et al., 2008; Hsu et al., 2004; Kim et al., 2017; Lam & Chua, 2002; Liu et al., 2004; Maganga et al., 2014; Memish et al., 2013b; Nkoghe et al., 2005; Oboho et al., 2015; Okware et al., 2002; Paton et al., 1999; Rahman et al., 2012; Sazzad et al., 2013; Schieffelin et al., 2014; Shen et al., 2004; Shuaib et al., 2014; Wang et al., 2004; Wilder‐Smith et al., 2005; Yu et al., 2004).

### 2.3 Data extraction and summarising

We considered only the field reports containing bat‐originated zoonotic diseases for data abstraction. The extracted data included the country and location of the outbreak, year of sample, total sample, total death and possible associating factors such as causal agent, transmission dynamics, pathogenicity etc.

### 2.4 Data analysis

All of the extracted data were organised in a Microsoft Excel spreadsheet and then analysed using STATA/IC‐13.0 (Stata Corp, 4905 Lakeway Drive, College Station, Texas 77845 USA). Crude estimation of case fatality rate was performed by dividing the number of deaths of humans by the total number of affected and expressing the results as percentages. The crude estimate of case fatality rates, the 95% confidence interval (CI) and the *p* value were calculated on different diseases between Asia and Africa. Variations among the studies were evaluated using the chi‐square (χ^2^) test on Cochran's *Q* statistics (with *p* value) followed by *I* ^2^ statistics to determine the degree of heterogeneity in the study. The weights were chosen to reflect the amount of information that each study contains. A random‐effect meta‐analysis was applied using the ‘metan’ command specifying the random effects due to the high degree of heterogeneity (*I* ^2^ > 75%). The outputs have been illustrated using a forest plot (Higgins & Thompson, 2002).

## 3 RESULT

### 3.1 Demographic characteristics of different studies

**FIGURE 2:**
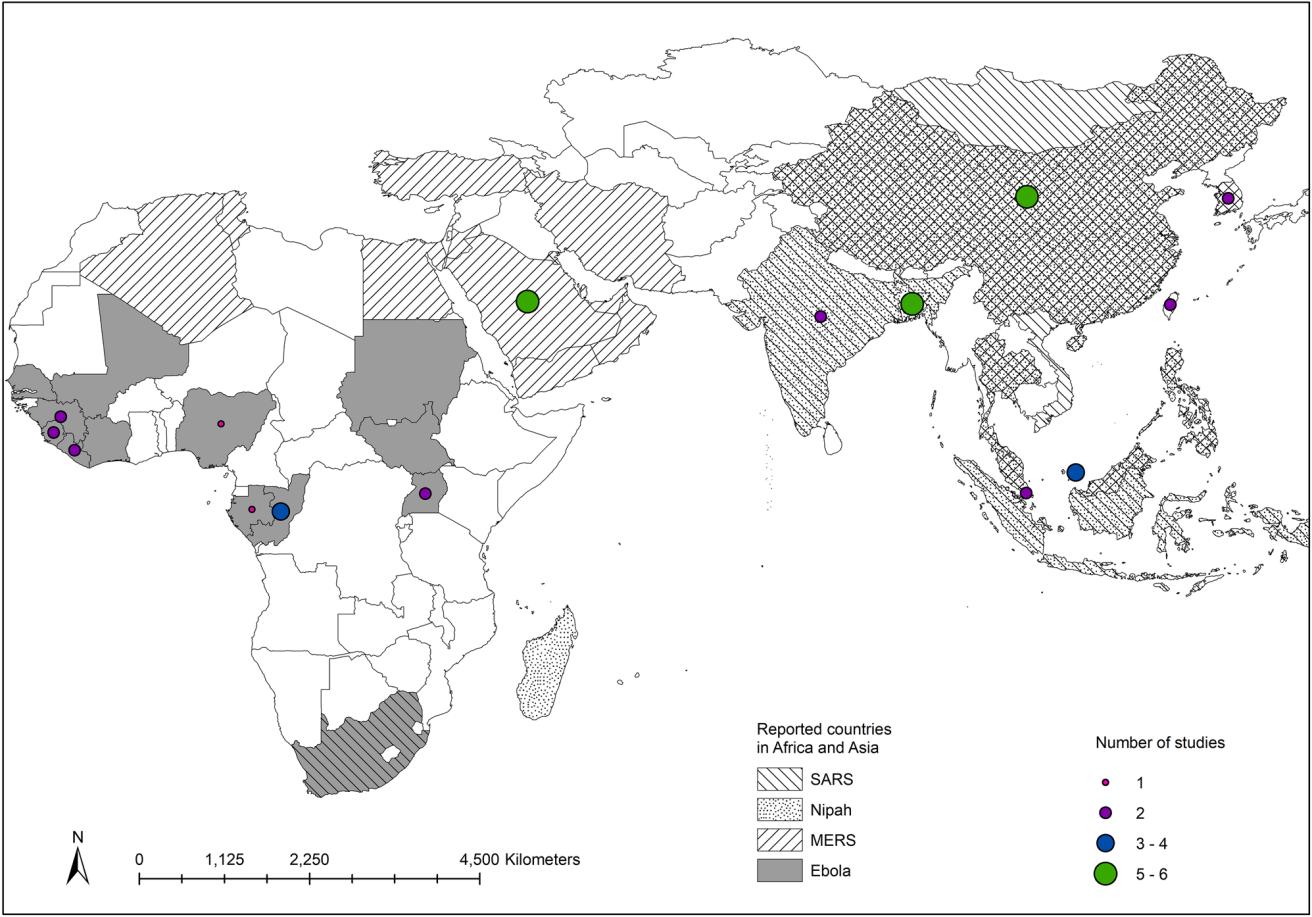
Reported countries (Asia and Africa) of Nipah, MERS, SARS and Ebola with number of studies conducted showed in the global map

We reviewed 41 published articles of the last two decades (1999–2021) that reported bat‐originated major zoonotic diseases, particularly in Asia and Africa (Table 1 and Figure 2). In this meta‐analysis, 73.17% of studies included from Asia and most of them are from China (14.63%), Bangladesh (12.20%), Malaysia (9.76%) and South Korea (9.76%), respectively. In Middle Eastern countries, Saudi Arabia (12.20%) mostly convicted with MERS (21.95%), which is a similar type of the SARS virus (21.95%). However, in published articles, Nipah (26.83%) is considered as the dominant virus for the South‐Asiatic region likely in Malaysia, Bangladesh and Taiwan, whereas Ebola (29.27%) is pre‐dominant for African zone likely in Democratic Congo (7.32%), Uganda (4.88%) and Nigeria (2.44%).

**TABLE 1:** The study characteristics included in the review (*N* = 41)

Characteristics	Frequency (%, 95%CI)	References
Publication year		
1999–2005	19 (46.34%, 30.66–62.58)	(Chen et al., 2004; Cheng et al., 2004; Chong et al., 2002; Chua et al., 1999; Ding et al., 2003; Dowell et al., 1999; Francesconi et al., 2003; Goh et al., 2000; He et al., 2005; Hsu et al., 2004; Lam & Chua, 2002; Liu et al., 2004; Nkoghe et al., 2005; Okware et al., 2002; Paton et al., 1999; Shen et al., 2004; Wang et al., 2004; Wilder‐Smith et al., 2005; Yu et al., 2004)
2006–2015	15 (36.59%, 22.12–53.06)	(Ajlan et al., 2014; Bah et al., 2015; Chadha et al., 2006; Chen et al., 2004; Christie et al., 2015; Cowling et al., 2015; Dixon et al., 2014; Homaira et al., 2010; Hossain et al., 2008; Maganga et al., 2014; Memish et al., 2013b; Oboho et al., 2015; Rahman et al., 2012; Sazzad et al., 2013; Schieffelin et al., 2014; Shuaib et al., 2014)
2016–2021	7 (17.07%, 7.15–32.06)	(Ahmed, 2017; Alshukairi et al., 2018; Aruna et al., 2019; Arunkumar et al., 2019; Bah et al., 2015; Cho et al., 2016; Choe et al., 2017; Christie et al., 2015; Cowling et al., 2015; Kim et al., 2017; Oboho et al., 2015)
Diseases		
SARS	9 (21.95%, 10.56–37.61)	(Chen et al., 2004; Cheng et al., 2004; Ding et al., 2003; He et al., 2005; Liu et al., 2004; Shen et al., 2004; Wang et al., 2004; Wilder‐Smith et al., 2005; Yu et al., 2004)
MERS	9 (21.95%, 10.56–37.61)	(Ahmed, 2017; Ajlan et al., 2014; Alshukairi et al., 2018; Cho et al., 2016; Choe et al., 2017; Kim et al., 2017; Memish et al., 2013b; Nkoghe et al., 2005; Oboho et al., 2015; Schieffelin et al., 2014)
Ebola	11 (26.83%, 14.22–42.94)	(Aruna et al., 2019; Bah et al., 2015; Christie et al., 2015; Dixon et al., 2014; Dowell et al., 1999; Francesconi et al., 2003; Maganga et al., 2014; Nkoghe et al., 2005; Okware et al., 2002; Schieffelin et al., 2014; Shuaib et al., 2014)
Nipah	12 (29.27%, 16.13–45.54)	(Arunkumar et al., 2019; Chadha et al., 2006; Chong et al., 2002; Chua et al., 1999; Goh et al., 2000; Homaira et al., 2010; Hossain et al., 2008; Hsu et al., 2004; Lam & Chua, 2002; Paton et al., 1999; Rahman et al., 2012; Sazzad et al., 2013)
Continents		
Asia	30 (73.17%, 57.06–85.78)	(Ahmed, 2017; Ajlan et al., 2014; Alshukairi et al., 2018; Arunkumar et al., 2019; Chadha et al., 2006; Chen et al., 2004; Cheng et al., 2004; Cho et al., 2016; Choe et al., 2017; Chong et al., 2002; Chua et al., 1999; Cowling et al., 2015; Ding et al., 2003; Goh et al., 2000; He et al., 2005; Homaira et al., 2010; Hossain et al., 2008; Hsu et al., 2004; Kim et al., 2017; Lam & Chua, 2002; Liu et al., 2004; Memish et al., 2013b; Oboho et al., 2015; Paton et al., 1999; Rahman et al., 2012; Sazzad et al., 2013; Shen et al., 2004; Wang et al., 2004; Wilder‐Smith et al., 2005; Yu et al., 2004)
Africa	11 (26.83%, 14.22–42.94)	(Aruna et al., 2019; Bah et al., 2015; Christie et al., 2015; Dixon et al., 2014; Dowell et al., 1999; Francesconi et al., 2003; Maganga et al., 2014; Nkoghe et al., 2005; Okware et al., 2002; Schieffelin et al., 2014; Shuaib et al., 2014)
Country		
Bangladesh	5 (12.20%, 4.08–26.20)	(Homaira et al., 2010; Hossain et al., 2008; Hsu et al., 2004; Rahman et al., 2012; Sazzad et al., 2013)
China	6 (14.63%, 5.57–29.17)	(Cheng et al., 2004; Ding et al., 2003; He et al., 2005; Liu et al., 2004; Shen et al., 2004; Yu et al., 2004)
Taiwan	2 (4.88%, 0.06–16.53)	(Chen et al., 2004; Wang et al., 2004)
India	2 (4.88%, 0.06–16.53)	(Arunkumar et al., 2019; Chadha et al., 2006)
South Korea	4 (9.76%, 0.27–23.13)	(Ajlan et al., 2014; Cho et al., 2016; Choe et al., 2017; Cowling et al., 2015; Kim et al., 2017)
Malaysia	4 (9.76%, 0.27–23.13)	(Chong et al., 2002; Chua et al., 1999; Goh et al., 2000; Lam & Chua, 2002)
Saudi Arab	5(12.20%, 4.08–26.20)	(Ahmed, 2017; Ajlan et al., 2014; Alshukairi et al., 2018; Memish et al., 2013b; Oboho et al., 2015)
Singapore	2 (4.88%, 0.06–16.53)	(Paton et al., 1999; Wilder‐Smith et al., 2005)
Gabon	1 (2.44%, 0.06–12.86)	(Nkoghe et al., 2005)
DR Congo	3 (7.32%, 1.54–19.92)	(Aruna et al., 2019; Dowell et al., 1999; Maganga et al., 2014)
Uganda	2 (4.88%, 0.06–16.53)	(Francesconi et al., 2003; Okware et al., 2002)
Sierra Leone	1 (2.44%, 0.06–12.86)	(Schieffelin et al., 2014)
Liberia	1 (2.44%, 0.06–12.86)	(Christie et al., 2015)
Guinea	1 (2.44%, 0.06–12.86)	(Bah et al., 2015)
Nigeria	1 (2.44%, 0.06–12.86)	(Shuaib et al., 2014)
Guinea + Liberia + Sierra Leon	1 (2.44%, 0.06–12.86)	(Dixon et al., 2014)

### 3.2 Meta‐analysis of case fatality rates by the selected diseases

**FIGURE 3:**
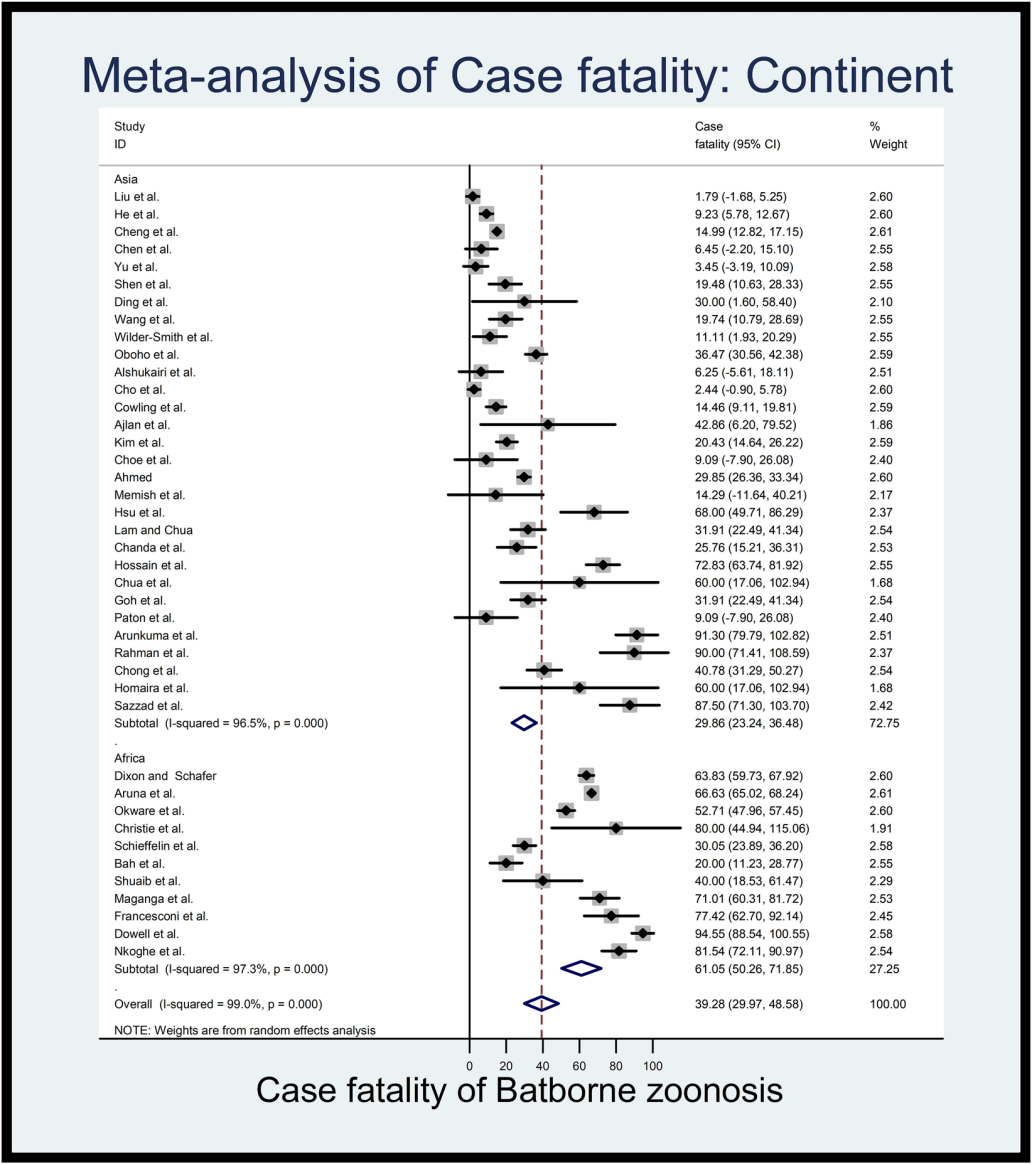
Forest plot of the estimated case fatality rates of bat‐originated viral zoonotic diseases in Asian and African countries (the centre dot representing point estimates whereas grey square representing the weight of each study to the meta‐analysis)

Based on the available data, the estimated pooled case fatality rates of the different types of bat‐originated zoonotic viral diseases of humans in Asia and Africa have been presented in Table 2. The random effect of the meta‐analysis showed that the pooled case fatality rates of bat‐originated viral zoonotic diseases were 29.86 in Asia (95%CI: 23.24 to 36.48, *l* ^2^% = 96.5, *p *< 0.001) and 61.06% in Africa (95%CI: 50.26 to 71.85, *l* ^2^% = 97.3, *p *< 0.001). Figure 3 showed the estimates case fatality rates from individual studies on continents, which ranged from 1.79% (95% CI: –1.68 to 5.25) to 90% (95% CI: 71.41 to 108.59) in Asia and 20% (95% CI: 11.23 to 28.77) to 94.55% (95%CI: 88.54 to 100.55) in Africa with an overall estimated case fatality rate of 39.28% (95%CI: 29.97 to 48.58, *l* ^2^% = 99.0, *p *< 0.001).

**TABLE 2:** Estimated pooled case fatality rates of bat‐originated major zoonosis in Asia and Africa

World region	Pooled estimates %	95%CI	Heterogeneity chi‐squared (*χ* ^2^)	*l* ^2^%	*p* Value
Asia	29.86	23.24–36.48	817.69	96.5	<0.001
Africa	61.06	50.26–71.85	368.66	97.3	<0.001

CI: confidence interval; *I* ^2^: inverse variance index; *χ* ^2^: Cochran's Q chi‐square.

**FIGURE 4:**
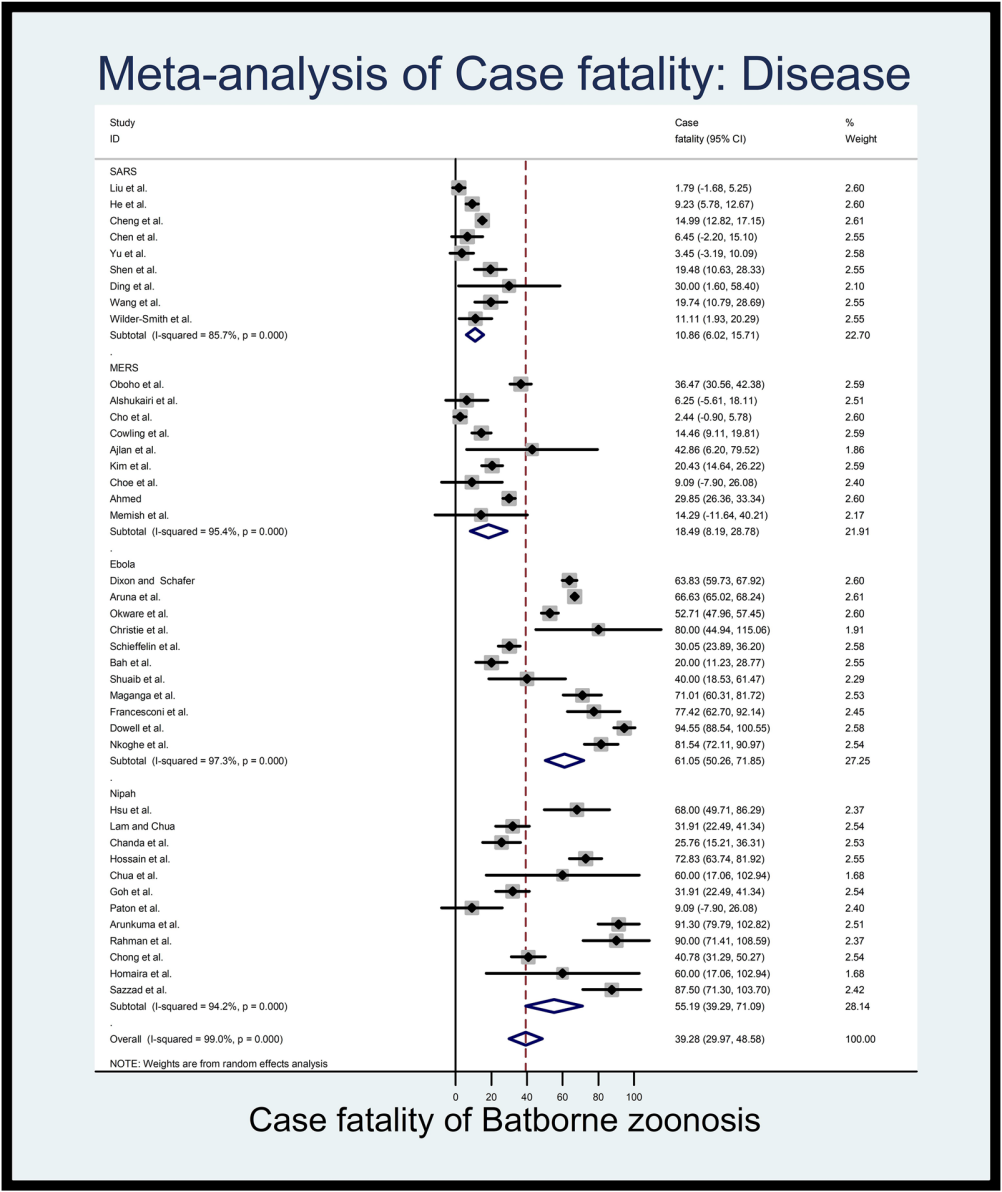
Forest plot of the estimated case fatality rates of bat‐originated viral zoonotic diseases in human (the centre dot representing point estimates whereas grey square representing the weight of each study to the meta‐analysis)

The estimated pooled case fatality rates for bat originates viral zoonotic disease are presented in Table 3. The highest estimated case fatality rate was for Ebola (61.06%; 95%CI: 50.26 to 71.85, *l* ^2^% = 97.3, *p *< 0.001) followed by Nipah (55.19%; 95%CI: 39.29 to 71.09, *l* ^2^% = 94.2, *p *< 0.001), MERS (18.49%; 95%CI: 8.19 to 28.76, *l* ^2^% = 95.4, *p *< 0.001) and SARS (10.86%; 95%CI: 6.02 to 15.71, *l* ^2^% = 85.7, *p *< 0.001). Figure 4 shows the estimates of case fatality rates from individual studies of viral zoonotic diseases in humans originating from bats, which ranged from 1.79 (95%CI: –1.68 to 5.25) to 30 (95%CI: 1.60 to 58.40) for SARS, 2.44 (95%CI: –0.90 to 5.78) to 42.86 (95%CI: 6.70 to 79.52) for MERS, 20 (95%CI: 11.23 to 28.77) to 94.55 (95%CI: 88.54 to 100.55) for Ebola and 9.09 (95%CI: –7.90 to 26.86) to 91.30 (95%CI: 79.79 to 102.82) for Nipah encephalitis. Moreover, the overall estimated case fatality rate of viral zoonosis in human from individual studies are 39.28% (95%CI: 29.97 to 48.58, *l* ^2^% = 99.0, *p *< 0.001).

**TABLE 3:** Estimated pooled case fatality rates of different bat‐originated major zoonosis

Disease	Polled estimates %	95%CI	Heterogeneity chi‐squared (χ^2^)	*l* ^2^%	*p* Value
SARS	10.86	6.02–15.71	56.02	85.7	<0.001
MERS	18.49	8.19–28.76	174.24	95.4	<0.001
Ebola	61.06	50.26–71.85	368.66	97.3	<0.001
Nipah	55.19	39.29–71.09	190.53	94.2	<0.001

CI: confidence interval; *I* ^2^: inverse variance index; χ^2^: Cochran's Q chi‐square.

## 4 DISCUSSION

### 4.1 Case fatality rates of the selected viral diseases

Our estimated case fatality rate (CFR) of Ebola virus disease (EVD) were similar to the CFR of the reported review (65.4%), and WHO also reported that CFR around 50% in the infected areas (Rugarabamu et al., 2020). Different reports identified the case fatality rate as 40% to 75% for this virus spillover. Similarly, the estimated CFR of Nipah virus infection was suggested in another study to be where approximately 50%–75% of those infected ended up deceased (Arunkumar et al., 2019). Nipah virus outbreak was first recognised among pig farmers in Malaysia. The case fatality rates detected in humans might depend on the capabilities of epidemiological surveillance system and clinical management (Lam & Chua, 2002). Out of 265 human cases, 105 were fatal, leading to the culling of 1 million pigs to contain the outbreak (Ang et al., 2018). An outbreak was subsequently reported in Bangladesh with annual outbreaks occurring, and periodic outbreaks being observed in India as well (Epstein et al., 2020). Other regions may be at risk for infection as the virus has been found in its natural reservoir host (*Pteropus* bat species) in several countries, including Cambodia, Indonesia, Thailand, Philippines, Ghana and Madagascar (Olival et al., 2020b).

In addition, the SARS epidemic was first observed in June 2003. The incidence was attributed with 8422 cases, with a case fatality rate of 11% (Morens & Fauci, 2020). The virus is considered to be eradicated, but the capabilities of this virus to infect animals are likely to re‐emerge in the near future. Out of 7 coronaviruses, the SARS‐CoV‐2, SARS‐CoV and MERS‐CoV can lead to severe respiratory syndromes, with about 6.76%, 9.6% and 35.5% mortality rates, respectively. A whole‐genome scan has shown that SARS‐CoV‐2 has a 79% similarity to SARS‐CoV and a 50% similarity to MERS (Hu et al., 2020). All previous data have demonstrated similar findings to our study.

### 4.2 The initial and all bat‐born virus spillover confined in Asia and Africa

Growing urbanisation, deforestation, human expansion and habitat loss are the risk factors for inducing virus spillover. The tropical regions, particularly in Asia and Africa, have been recognised as being rich in host biodiversity and as having large pool of pathogens that increases the chance of a novel pathogen to emerge. Throughout Africa and Asia, the intensive farming system provides a unique opportunity that has a key role in how emerging and re‐emerging pathogens could emerge and spread between species (Hassell et al., 2017). The overburdened population has been involved in Bushmeat activities, such as hunting and butchering, which are particularly widespread in sub‐Saharan Africa. This threatens animal diversity, fragments the landscape and, significantly, impacts the overall ecological balance. Bushmeat has been identified as the primary cause of the Ebola outbreak in the African continent (Kurpiers et al., 2016). The long‐standing culture to consume raw and fresh date palm sap in rural areas in Bangladesh has continuously posed a threat of Nipah outbreaks in the epidemic pattern since 2001.

Traditional Chinese medicine used to treat illnesses like asthma, arthritis, epilepsy and erectile dysfunction (Calisher et al., 2006) provides an avenue to cross the species barrier of deadly viruses from bat to human. Different wild animal species such as tigers, bears, rhinos, pangolins and other wild animal species are poached to mix their body parts to make these herbal medications. Thus, the Chinese traditional medication system can be a contributor to increasing animal–human interactions in this part of the world and a possible source of future pandemics (Still, 2003).

### 4.3 How current pandemics have evolved through the virus spillover from wildlife, possibly bats to humans

The spillover of bat‐originated viruses provides a significant number of insights for forecasting pandemics. Bats are a suitable reservoir for hosting 60 zoonotic pathogenic viruses, that transform this unique mammalian host to a potential hub for spillover and spillback for zoonotic viruses over the last few decades (Naicker, 2011). The diversity, richness and abundance of viruses in this host species, encourage the researcher/scientist to study intensely around this species in order to predict future pandemics. In this meta‐analysis, we revealed that phylogenetically resembled MERS and SARS viruses demonstrating the benchmark for the ongoing coronavirus infection through SAR‐CoV‐2. These coronaviruses (SARS, MERS) were silently evolving within wildlife species (possibly bats), in the past few decades. These viruses had the potential to become a pandemic in the past through different spillover events across in Asia and Africa (Table 1). The question is how they evolve in hosts like bats and other wildlife in its niche. The physiological system of bats is more unique than other animals and birds (Hossain et al., 2013a, 2013b) and provides an optimum niche for these viruses to reproduce rapidly and attain exclusive spillover ability without any clinical diseases in this host (Islam et al., 2020). They could mitigate the viruses within their delicate immune system, but unfortunately, the species jumping through bats to humans induce the virus to overwhelm the human immune mechanism.

Nipah virus, the genus of the Henipavirus, belongs to the paramyxoviridae family and was first isolated from pigs in 1999 in a Malaysian outbreak (Eaton et al., 2006). The countries of Southeast Asia are a hub of bat diversity, comprising 13 species of Megachiroptera and 60 species of Microchiroptera (Calisher et al., 2006; Islam et al., 2015). An adult Chinese pig farmer who was in direct contact with live pigs developed clinical signs of fever and encephalitis with respiratory illness. The large (*Pteropus vampyrus*) and small (*Pteropus hypomelanus*) flying foxes were found to be the natural reservoir hosts for the Nipah virus in the post‐outbreak surveillance by the Malaysian authority (Kulkarni et al., 2013). Since 2001, the Northeastern districts of Bangladesh have noticed sporadic outbreaks of Nipah virus‐associated disease in humans (Anderson et al., 2019; Epstein et al., 2020; Rahman et al., 2021). Although these outbreaks had many similarities to those of the Malaysian outbreak, there were no intermediate hosts in the Bangladesh outbreak with evidence suggesting human‐to‐human transmission. Serologic assay performed through utilising domestic and wild animals in the outbreak areas during 2001 and 2003 suggests the presence of Nipah virus antibody in Indian flying foxes (Epstein et al., 2020; Singh et al., 2019). Nipah virus or a closely related virus, notably Hendra virus, was widespread across the range of Indian flying foxes, with this being attributed to the occurrence of Nipah virus infections in humans in India in 2001 (Kulkarni et al., 2013). Hendra and Nipah viruses have long been circulating in natural hosts and altered foraging and behavioural patterns and the expansion of viral diversity, which increases their proximity to humans and livestock species (Islam et al., 2016).

In 2003, clusters of ‘atypical pneumonia’ were reported in Guangdong Province of China, subsequently spreading to Hong Kong. Then a novel CoV virus (SARS‐CoV) (Weiss & Navas‐Martin, 2005) was isolated that was later renamed as Severe Acute Respiratory Syndrome (SARS) (Cheng et al., 2007). This virus was transmitted from Hong Kong to the rest of the world through international travel, and over 8000 people in 26 countries became infected, with a high case fatality rate. SARS posed a severe public health threat to the world, with a significant negative impact on the global economy. This virus originated from intermediate horseshoe bats (*Rhinolophus affinis*), and subsequently, inter‐species transmission to humans took place via an intermediate host (Song et al., 2005). This virus is a distant relative of the coronavirus family, named SARS‐CoV (Shi & Hu, 2008), which infects many animal species, including rodents, cattle, dogs, pigs and humans (Shi & Hu, 2008). It is distinctly from two other coronaviruses recently identified in bats in southern China of the Asian continent. Epidemiologic studies and surveys provide evidence that SARS‐CoV‐like viruses were from masked palm civets (*Paguma larvata*) and raccoon dogs (*Nyctereutes procyonoides*) (Song et al., 2005).

In addition, SARS‐CoV‐like virus antibodies were detected in a hog badger (*Arctonyx collaris*) in a wildlife market in Shenzhen, China. The genomes of SARS‐CoV isolates from civets (Shi and Hu, 2008) and humans during the outbreaks of SARS in Asia, phylogenetically, are closely related to the group of SARS‐CoV‐like viruses that circulate within bats of this part of the world. SARS‐CoV‐like coronavirus viruses (Calisher et al., 2006) were detected in horseshoe bat species (*Rhinolophus* spp.), including *R. sinicus, R. ferrumequinum, R. macrotis, R. pearsoni* and *R. pusillus* and have transmitted in the meat markets to amplifying hosts, such as masked palm civets, raccoon dogs and a hog badger (Ge et al., 2016; Lau et al., 2005). The first human spillover was from them, in close contact with bats. Subsequent human‐to‐human transmission happened with adaptive mutations in the viral genome (Calisher et al., 2006). This SARS virus (like the new coronavirus) had infected humans in Saudi Arabia via a bat to camel pathway in 2012, infecting human respiratory systems, similar to SARS renamed as MERS (Al‐Osail & Al‐Wazzah, 2017). MERS hit the Middle East countries, but serological evidence of infections in camels were also detected in Pakistan and Bangladesh (Islam et al., 2018; Saqib et al., 2017). A total of 27 countries were affected by MERS during the outbreaks, spanning the Middle East (Saudi Arabia 80%, Egypt, Oman, Qatar, Jordan, Kuwait) and other parts of the world, including Europe, Asia and North America (Ramadan & Shaib, 2019). As a zoonotic case, MERS‐CoV is primarily initiated in bats, and the primary reservoir host is the dromedary camel, which is the animal source of infection in humans (World Health Organization, 2019). The natural cases of MERS are sporadic; however, the human‐to‐human secondary type transmission is widespread, mostly at household and nosocomial levels. Most MERS cases have been identified in health care workers and among their family members due to inadequate infection control or unhygienic caring practices of the infected patients (WHO, 2018). The MERS‐CoV antibodies have been detected in camels of Bangladesh and more recently in Pakistan (Islam et al., 2018).

Another bat‐borne virus, namely Ebola virus, was detected in terrestrial mammals in the Central African Republic and later antibodies identified in three fruit bat species (*Rousettus leschenaultii*, *Cynopterus* spp., *Megaderma lyra*) of the EPteropodidae family. These infected bats have been recognised in Bangladesh (Olival et al., 2013), and about 3.5% of them were serologically positive for antibodies against *Ebola zaire* and *Reston viruses*. *Zaire Ebola viruses*, *Bundibugyo Ebola viruses*, *Taї forest Ebola viruses* and *Sudan Ebola viruses* are the four Ebola viruses that have the potential to infect humans. Their geographical distribution is mostly confined to Central and East Africa, and South Africa, with high case fatality rates ranging between 53% and 88% (Muyembe‐Tamfum et al., 2012). During the 2013–2016 West African outbreaks, the virus originated in Guinea and then crossed geographical boundaries, affecting at least six African countries and more than three urban settings This highlights the importance of surveillance and prediction for future pandemics (World Health Organization, 2015). Non‐human primates in contact with bats, including chimpanzees, apes and monkeys, maintained the transmission cycle (Rewar & Mirdha, 2014). In humans, the disease may be spread by handling the bushmeat (wild animals hunted for food) and from other animals who have been in contact with infected fruit bats, non‐human primates and forest antelope (Rewar & Mirdha, 2014). However, people are initially infected through direct contact with the blood, skin or bodily fluids of sick Ebola patients, contact with animals or horizontally by individuals who have died from the disease (Centers for Disease Control and Prevention, 2019).

### 4.4 Bats unique immune system pose the threat of virus spillover events more frequently

The immune system of bats has been tailor made, allowing them to be able to evade the pathogenic effect, for a diverse range of viruses within their physiological system. This unique flying mammal has evolved their physiological processes to resist deadly pathogenic microorganisms and has an incredible ability to limit the inflammation phenomena being elicited by pathogens like MERS and SARS (Subudhi et al., 2019). Bats have an evolutionary physiological trick involving the secretion of interferon‐alpha, initiating rapid immune responses, which signal to other cells throughout the body to minimise inflammation and immune protection from viruses (Subudhi et al., 2019). By accelerating the metabolic rates, these animals can generate extreme body heat, which mitigates the infectivity of the viruses in their body. In general, vigorous physical activity attained through flying and a high metabolic pathway assists bats in overcoming tissue damage due to the accumulation of reactive molecules, primarily free radicals. Besides, to enable flight, bats physiological systems been evolved to mop up destructive and highly reactive molecules efficiently and effectively. These phenomena have been attributed to their uniquely long lifespans and the harbouring of these deadly viruses.

### 4.5 SARS and MERS continuously being exposed to human respiratory systems, possibly an avenue for the ongoing pandemic

The ongoing COVID‐19 virus (Decaro & Lorusso, 2020) is detrimental to human respiratory systems, although it has been imprinted in the nervous and blood vascular system as well. The other coronaviruses like MERS and SARS have an extreme affinity to the airway passage in humans. These two viruses have developed our ability to better understand respiratory system physiology. In contrast, Ebola and Nipah virus have the affinity to infect nervous systems through inducing neurological symptoms in affected individuals. These coronaviruses (MERS, SARS) are well adapted and well equipped to evade the immune system by inducing spillover in the Asian parts of the world; thus, these viruses have evolved in bats and other wildlife over the past few decades. In addition, wildlife species and human interaction in the context of deforestation and increased population growth in these regions (Asia and Africa), triggers a mighty force of species jumping events more frequently. The recent SARS‐CoV‐2 virus has undergone widespread antigenic changes from its beginning, leading to the emergence of a new type of coronavirus (CoV), now known as COVID‐19, which is immunologically different from the previous circulating viruses such as MERS and SARS. The COVID‐19 virus rapidly gained the ability to utilise the angiotensin‐converting enzyme‐2 (ACE‐2) (Tay et al., 2020), which is an essential receptor on the host (human) cell membrane, and its interaction with spike protein (SP) with a furin‐cleavage site, resulting in the SARS‐CoV‐2 invasion into human respiratory cells in a more efficient way. The SARS‐Cov‐2 has similarities with SARS CoV in tissue tropism but has dissimilarities suggesting an invasion of this SARS CoV‐2 in an intermediate host (Andersen et al., 2020). However, differences between the bat coronavirus and SARS‐CoV‐2 suggest that humans were infected via an intermediate host, such as pangolins (Zhao et al., 2020). The source of viral transmission to humans remains unclear, but undoubtedly, the virus was evolving in the past few years producing a global pandemic.

### 4.6 The distribution of bats across the Asian and African continent elicits the virus spillover more realistically

Bats are the most abundant of mammals, and except for humans and perhaps rodents, they are the most widely distributed mainland mammals in Asia and Africa (Calisher et al., 2006). Bat populations may be panmictic or may exist as metapopulations (Calisher et al., 2006), offering the potential for seasonal, annual and periodic outbreaks as well. The population densities of bats in their roost and crowded roosting behaviour increase the intra‐ and interspecies transmission of viral infections. In addition, the potential for both migratory and non‐migratory populations serves as triggering factors for this transmission through spillover and spillback mechanisms. A recent preprint article describes the risk of potential future outbreaks of coronaviruses in Southeast Asia. This region of the world is massively populated, and the abundance of bat diversity is huge. This region, particularly, southern China, northeastern Myanmar, Lao PDR and northern Vietnam, has the highest diversity of SARS‐CoV bat host species (Sánchez et al., 2021). A recently reviewed article demonstrated the possible expansion of reservoir hosts of ongoing SARS‐CoV‐2 virus, which puts public health at significant risk as the virus has infected immunologically naïve bats in a North American region (Olival et al., 2020a). Therefore, bat biology is a neglected and unprivileged issue, and it gives rise to a significant number of questions regarding the role of bats in disease emergence. Bats have played a unique role in a complex and delicate process that propagates and transmits viruses from bats to humans via an intermediate host (Afelt et al., 2018). The previous two coronaviruses (MERS, SARS) utilise the intermediate host for amplification to jump on to humans and, unfortunately, the ongoing SARS‐CoV‐2 can potentially infect humans directly (Sánchez et al., 2021). This characteristic distinguishes bats from all other mammals, which might contribute to increased assessment during a pandemic outbreak. Viruses that evolved within bat species might utilise replicating cellular receptors and biochemical pathways, which are conserved in mammals (Schountz, 2014). However, these conserved cellular receptors and pathways might enhance the transmission of bat‐associated viruses to other mammals, including humans. Bat demographic and spatial distribution is variable in providing opportunities for viruses that cause both acute and persistent infections to be maintained (Hayman et al., 2013).

### 4.7 Briefly describe the transmission cycle of those four viruses along with possible route of COVID‐19

**FIGURE 5:**
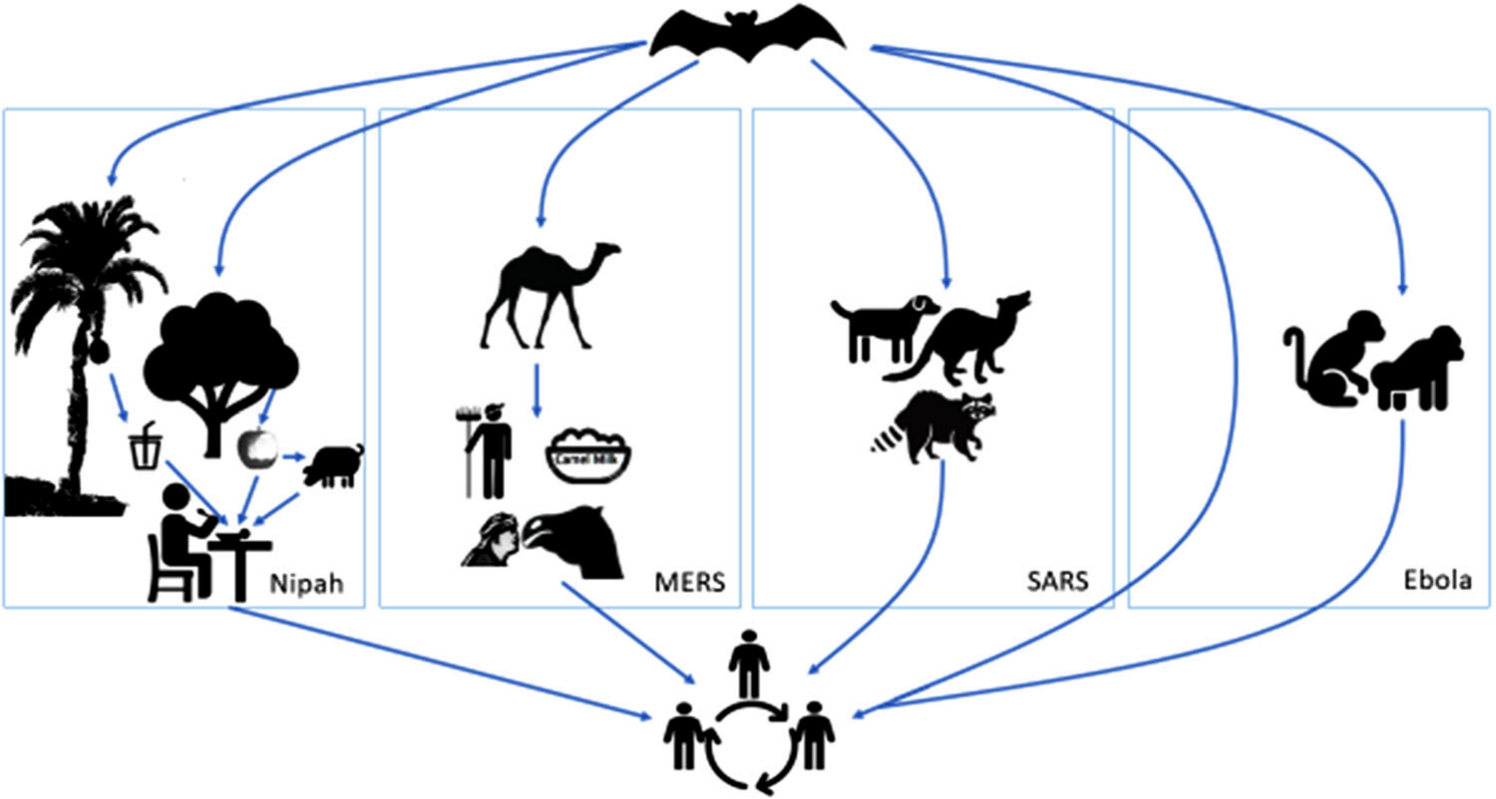
Transmission dynamics of Nipah, MERS, SARS and Ebola between bats and humans and further transmission among humans to humans through direct contact

MERS‐CoV was identified in bats (Li et al., 2017; Memish et al., 2013a), and the virus has been circulating in dromedary camels for over 35 years (Killerby et al., 2020). In camels, it could induce an asymptomatic infection or produce mild upper respiratory infection. Studies found that MERS‐CoV could be shared from camels mainly through the nasooral route. There is also evidence that MERS‐CoV RNA might be detectable in camel milk and faeces (Sikkema et al., 2019). Contact with a camel, which is shedding the virus, could be the source of human infection. The Arabian culture of kissing or hugging a camel, working on a camel farm and drinking unboiled/unpasteurised camel milk (Figure 5) increases the chance of humans getting infected with the virus (Farag, 2019; Killerby et al., 2020). Nipah transmission from bats to humans in South Asia, such as Bangladesh, is the contamination of raw date palm sap by bats, with subsequent consumption by humans. In East Asia, in countries such as Malaysia and India, the infection of domestic animals such as pigs happened most likely from ingesting foods contaminated with bat saliva or urine, with subsequent transmission to human. In the African countries, humans can get Ebola virus infection through close contact with infected animals, such as chimpanzees, fruit bats and forest antelope as well as humans. The major route of transmission of Ebola is from handling or consumption of bushmeat and contact with infected bats. Contact with blood, body fluid or the skin of infected animal/humans is a source of viral transmission (Rewar & Mirdha, 2014). The palm civet, a short‐legged carnivore, is the transmission host of SARS‐CoV from bat to humans (Fong, 2017) and we have limited evidence of person‐to‐person transmission of this virus (Luby et al., 2009). We have only described four major bat‐borne diseases in the context of their emergence in Asia and Africa; climate change and other environmental risk factors have not been the focus of this review.

## 5 CONCLUSION

Bat‐associated viruses have continued to evolve, and their interaction with humans allows them to cross the species barrier, eliciting future epidemics or pandemics. The ongoing COVID‐19 pandemic is the outcome of this evolving process that has long been incubating in wildlife species (possibly bats) and has developed its ability to infect humans in massive ways. The previous coronaviruses (MERS and SARS) have respiratory cellular tropism to allowing the ability to infect humans in continuing virus spillover. These phenomena further indicate that a more likely respiratory pathogen might evolve from wildlife, with the potentiality of causing a global pandemic. In addition, human behaviour, deforestation, wildlife trade and consumption, and the interaction with wildlife are the critical factors to transmitting viruses from bat to human. Human interactions with wildlife, especially hunting bats for meat, wildlife trade and the use of bat guano in the agricultural production system (all of which persist in Southeast Asian countries for a considerable period), pose a severe indication of an occurring future pandemic this part of the world. As the current pandemic has shown, an infectious disease that starts in one part of the world can spread globally in no time whatsoever. There is an urgent need for constructive conservation strategies to prevent deforestation and reduce wildlife–human interactions. A comprehensive global surveillance system to monitor the emergence of bat‐borne viruses would be an indispensable tool in helping us fight these deadly and terrifying epidemics and pandemics.

## FUNDING INFORMATION

The research did not receive any grant from the funding agencies.

## ETHICAL STATEMENT

No ethical approval was required as the current work is a review.

## AUTHOR CONTRIBUTIONS

Conceived and designed the research: MMH and SAK. Data collection and verification: MMH, SAK, AZT, AI and MAI. Wrote the paper and commented: MMH, SAK, AZT, AI, MMI and MAI. All the authors read and approved the manuscript for submission to the journal for publication.

### PEER REVIEW

The peer review history for this article is available at https://publons.com/publon/10.1002/vms3.835.

## Data Availability

NA.
